# Trends of Healthy Life Expectancy of the Elderly in China in 1994–2015: Revisiting From the Perspective of Morbidity Transition

**DOI:** 10.3389/fpubh.2021.774205

**Published:** 2022-01-05

**Authors:** Zhen Zhang, Junhan Dong, Chenyuan Zhao, Qiang Li

**Affiliations:** ^1^Institute of Population Research, School of Social Development and Public Policy, Fudan University, Shanghai, China; ^2^School of Sociology and Population Studies, Renmin University of China, Beijing, China; ^3^Population Research Institute, School of Social Development, East China Normal University, Shanghai, China

**Keywords:** health life expectancy, life expectancy, disability-free life expectancy, activities of daily living, morbidity transition, older people, China

## Abstract

Research on healthy life expectancy (HLE) in China has been fueled by a spate of new data sources and studies, yet no consensus is reached on the pattern of HLE changes and the underlying mechanism. This study examined the change of HLE in China over 20 years with long term national data. Health status, measured by activities of daily living, is combined with mortality to calculate the disability-free life expectancy by the Sullivan method. The results show that the HLE rose slower than life expectancy (LE) in 1994–2004, indicating morbidity expansion. However, in 2010–2015, the proportion of HLE to LE increased, manifesting morbidity compression. A counterfactual analysis further shows that health improvement has been increasingly important in increasing HLE in 2010–2015, despite the dominance of mortality decline. The findings suggest that morbidity can transition between compression, expansion and dynamic equilibrium over a long period due to different combinations of mortality and health improvements. Given the limited data in this study, whether and how morbidity transitions unfold in the future remains open and requires further research.

## Introduction

China's population is aging fast, due largely to fertility decline and modestly to mortality decline (1). The proportion of people aged 60 and over is forecast to rise from 18.7 to 34.6% in 2020–2050, far faster than Europe, the oldest continent, where the proportion of older people will rise from 25.7 to 34.95% (2, 3). During the same period, Meanwhile, remaining life expectancy at age 60 will rise from 19.1 to 23.1 years for men and from 22.5 to 25.6 years for women (2). The rapid growth of the older population accompanied by the steadily lengthening of lifespan implies a great demand for health care and social support, posing a huge challenge to China in the future. The health status of older people and its trends is crucial for China's successfully dealing with population aging.

Increased longevity is not simply equivalent to increased quality of life. Healthy life expectancy (HLE) was thus developed to account for mortality and morbidity into a single indicator (4). Because of its desirable properties, HLE is widely used to address whether observed increases in life expectancy are accompanied by decreases in morbidity (5, 6). By now, three hypotheses have been developed on health trends in aging population: (1) Compression of morbidity (7), which states that the period of morbidity would compress with the increase of life expectancy (LE) (2). Expansion of morbidity, Gruenberg (8) and Kramer (9) argued that the improvement of medical technology helped to reduce the mortality rate of certain diseases and increase the survival probability of unhealthy people, and therefore, as the life expectancy of the elderly increases, the period of illness and the period of disability are expanded (3). In the middle was the hypotheses “Dynamic equilibrium” proposed by Manton (10), suggesting that the prevalence of disability may increase, but the severity of disability would decrease, which leads to balanced changes in healthy and unhealthy life.

The above three hypotheses have their empirical supports, probably due to different research designs, such as longitudinal or cross-sectional data, countries, ages under study, social groups and health measurements (5, 6, 11–16). In China, evidence is mixed. While some studies support the compression of morbidity [e.g., Guo and Gu (17); Zeng et al. (18)], others reported evidence for the expansion of morbidity (19, 20).

To date, great efforts have been made to gain insight into the mechanism underlying the inconsistency of HLE trends. One effective approach has been to examine changes in HLE and LE in as many countries as possible, thereby drawing out general patterns of morbidity change. For example, the Global Burden Disease study (GBD) collected data on mortality and morbidity from around the world and found that most of 178 countries showed morbidity expansion from 1990 to 2010 (5). The current accumulation of empirical findings allows us to rethink the dynamics of morbidity.

Implicitly or explicitly, older people are assumed to follow a similar pattern of morbidity change, either expansion or compression. Efforts have been made in the pursuit of consistent evidence of change in disability and morbidity, but the lack of consensus on the trends in changes is disturbing [e.g., Robine and Michel (12)]. Nevertheless, different populations may be at different stages of the mortality and health transition (21) and, thus, exhibit different characteristics of morbidity trends. Health transition is a process that is assumed to occur in stages, with important causative factors shifting from one stage to another. As such, the health transition lacks singularity or simplicity in the means by which it was achieved (22), and understandably so does morbidity transition. For instance, an earlier study based on the GBD project found that morbidity, indicated by health-adjusted life expectancy, tended to increase as life expectancy rose to around 70 years, after which morbidity flattened for men and appeared to decrease for women (23). Yong and Saito (24) investigated the trend in healthy life expectancy in Japan using self-rated health to measure health status and reported that from 1986 to 2004, Japanese men and women experienced morbidity compression until 1995, when LE was 76 years for men and 83 years for women, respectively, followed by morbidity expansion.

Even in the same country, different factors usually lead to the health transition at different times. Various interventions that may reduce mortality or improve health statuses, such as public health policies or medical advances, are rarely introduced simultaneously. The resulting transition in mortality and morbidity will unfold at different rates over time. Therefore, different patterns of morbidity trends may be observed over a long period of time. For instance, using a longitudinal dataset in 2002–2014 and the measurement of Activities of Daily Living, Song et al. (25) found that China's elderly people first experienced morbidity compression, followed by the dynamic equilibrium. Li et al. (26) reported that the disability-free life expectancy of older people in Shanghai switched from compression to expansion in 1998–2013. Based on 23-year multi-wave data, Deeg et al. (27) calculated physically healthy life expectancy using self-reports of major chronic diseases and Instrument Activity of Daily living and reported fluctuations across the study period. All of these findings, including the two studies by Mathers et al. (23) and Yong and Saito (24) mentioned above, suggest that the pattern of change in morbidity may not be fixed over time, even for the same population, and that instead, morbidity can transition between different states.

According to the morbidity transition perspective, a longer observation period would be desirable as it would cover multiple stages of morbidity transition. However, given some limitations of survey data (e.g., poor representativeness, small size and low reliability), it remains unclear whether conclusions drawn from survey data can be applied to national trends in healthy aging. Therefore, to fill this knowledge gap, this study examined health trends from 1994 to 2015 based on data from nationally representative large-scale surveys in 1994 and 2004 and the population censuses in 2010 and 2015, thus constructing a complete picture of the evolution of healthy aging in China.

## Data and Methods

### Data and Measurement

Health data of the elderly were obtained from the National Sample Survey of Population Changes (NSSPC) in 1994 and 2004, the 2010 census and the 2015 National 1% Population Sample Survey (micro-census).

NBS conducted the NSSPC surveys in 1994 and 2004. Respondents to the survey were randomly selected from 31 provinces in mainland China, 124,114 older people aged 60 years or older were included in the 1994 sample and 152,055 in 2004. Health status was measured by a question on activities of daily living [ADL: (28)]. “Are you able to perform the following activities of daily living: eating, dressing, bathing and going to the toilet?” The answer could be “Yes, I am” or “No, I am not.” Older people who chose the second answer were considered unable to look after themselves. The 2010 and 2015 censuses collected health data by using a question.” Your health status is ____.” Answers can be one of “healthy,” “mostly healthy,” “unhealthy but able to take care of yourself” and "disabled.” We have combined the first three responses into one category for older people who can look after themselves, leaving the fourth response for older people who cannot look after themselves.

As the daily ability measures in 1994 and 2004 differ from those in 2010 and 2015, it is difficult to directly compare the estimated health indicators between these two periods. Therefore, this study first investigated changes in HLE from 1994–2004 to 2010–2015, respectively. The relative rates of change between the two periods were then compared after removing the effect of different scales in the morbidity question.

The life tables were calculated from mortality data collected in censuses and micro-censuses from 1982 onwards. Taking into account the errors in mortality data, we reassessed mortality rates for census and micro-census years with reference to the most recent estimates of mortality from national statistical offices, the United Nations (2) and other scholars (29, 30).

We start our estimations with mortality data from 1982, assuming that age-specific mortality follows an exponential change (decline), as it usually does (31). The assumption of exponential change is reasonable given that no extreme events occurred from 1982 to 2015. The age pattern of mortality decline is similar to the general pattern noted by Tuljapurka et al. (32), whereby the major declines in mortality occur first in infants and children, followed by young adults, the young elderly and the elderly in that order. We modeled age-specific death rates for 1994, 2004, 2010, and 2015 by targeting LE in 1994 and 2004 estimated by the UN and LE in 2010 and 2015 by the NBS and other scholars (29, 30). Based on the resulting age-specific mortality, abridged life tables were constructed for 5-year age groups ending with 85 years and over. See Supplementary Material for detailed tables.

### Methods

Sullivan (33) proposed estimating HLE by combining health status prevalence in cross-sectional survey data with period life tables. With the removal of the effect of age structure, the estimated HLE can be compared across populations or the same population at different points in time. The method applies to the case without transition probabilities (34). Because of its straightforward and succinctness, the Sullivan method is commonly used in the estimation of HLE. Besides, multistate life tables can be used to calculate the HLE. This method considers the dynamic changes between functional states and mortality in different health states (18). However, it is more demanding on data that are required to obtain multistate data of different individuals. In recent years, microsimulation and Bayesian methods have been increasingly applied to estimate HLE (34, 35).

In this study we used the classic Sullivan method, which is based on life tables and disability data collected in censuses and national level sample surveys. The prevalence of disability for each age group person-years into years with and without disability. The number of surviving person-years in the life table is multiplied by the proportion of people able to care for themselves to give the number of surviving person-years without disability, which is then divided by the number of survivors to obtain an estimate of HLE.

As mentioned earlier, the HLE contains information on mortality and morbidity, so it derives its change from changes in mortality and health status. Therefore, counterfactual analysis is used to assess the contribution of mortality and health status improvements to changes in the HLE over time. For example, we will estimate a counterfactual HLE with the same health status as in 1994 but with a mortality rate of 2004. The difference between this HLE and the actual HLE can therefore indicate how much of the change in the HLE from 1994 to 2004 is due to a reduction in mortality. The contribution of health improvement is estimated by subtracting the change due to mortality decline from the actual change in HLE.

The results of the counterfactual analysis allow a comparison of changes in morbidity over time and the contribution of its components. This comparison can help determine whether morbidity is compressing or expanding, or whether a shift in morbidity is occurring.

## Results

### The 1994–2004 Period

#### The Trajectory of Healthy Life Expectancy of the Elderly in China

From 1994–2004, older people in China enjoyed an increase in life expectancy (Table 1). The LE for men rose by 0.20–1.05 years and for women by 0.26–0.70 years. For both men and women, the increase in LE decreases with age. The gender gap in gains in LE becomes smaller at higher ages, indicating a converging trend in mortality improvement.

**Table 1:** Disability-free life expectancy of the elderly, 1994 and 2004.

**Male**									
		**LE**			**HLE**			**HLE/LE (%)**	
Age	1994	2004	Δ	1994	2004	Δ	1994	2004	Δ
60	16.18	17.23	+1.05	14.99	15.69	+0.71	92.64	91.09	−1.55
65	12.63	13.48	+0.85	11.42	11.96	+0.55	90.42	88.79	−1.63
70	9.61	10.18	+0.57	8.38	8.69	+0.31	87.15	85.32	−1.83
75	7.30	7.63	+0.33	6.03	6.13	+0.10	82.69	80.41	−2.28
80	5.50	5.81	+0.31	4.23	4.28	+0.05	76.86	73.60	−3.26
85+	4.05	4.24	+0.19	2.91	2.84	−0.06	71.82	67.05	−4.78
**Female**									
		**LE**			**HLE**			**HLE/LE (%**)	
Age	1994	2004	Δ	1994	2004	Δ	1994	2004	Δ
60	19.04	19.69	+0.65	16.97	17.25	+0.29	89.13	87.63	−1.50
65	15.21	15.73	+0.51	13.13	13.32	+0.19	86.29	84.72	−1.57
70	11.71	12.13	+0.41	9.63	9.76	+0.13	82.19	80.50	−1.69
75	8.83	9.14	+0.31	6.77	6.84	+0.07	76.63	74.79	−1.83
80	6.44	6.70	+0.26	4.46	4.40	−0.07	69.38	65.66	−3.72
85+	4.66	4.91	+0.26	2.88	2.78	−0.10	61.86	56.59	−5.26

Δ, *the change in the indicator in 1994–2004*.

Over the same period, HLE increased in the younger elderly, but decreased in the oldest old. Gender differences were evident, with the increase in HLE for men at 60–74 years roughly twice that of HLE for women. For men aged 85+ and women aged 80+, the change in HLE ranged from an increase to a decrease due to poor performance in improving the health of the oldest people.

Women had higher LE and HLE than men, except for age 85+, though with a decreasing gap with age. But the ratio of HLE to LE is higher for men than for women, indicating that women had relatively poor health despite their longevity. Importantly, in 1994–2004, HLE rose less than LE or even decreased above age 80, and consequently, the ratio of HLE to LE declined, implying an expansion of morbidity. Noteworthy, for younger old, men experienced a greater decrease in the ratio of HLE to LE than women. However, for the oldest old, the ratio of HLE to LE for women declined by 1.4 percentage points more compared with men, suggesting that women are becoming less healthy at advanced ages.

#### The Effect of Mortality and Disability-Free Rate on the Changes in HLE

To determine the impact of mortality and disability-free rate, we conducted a counterfactual analysis. We calculated a hypothetical HLE using the 2004 life table and the 1994 disability-free rate (DFR) and compared it to the actual HLE in 1994. As the two HLEs share the same DFR, their differences indicate the effect of mortality changes.

The results of the counterfactual analysis are presented in Table 2, where columns (1), (2) and (4) are the same as the three columns in the middle of Table 1. Here column (5) is obtained by subtracting column (3) from column (1) and represents the counterfactual change in HLE that would have occurred if the DFR had remained the same as in 1994 and only the mortality rate had changed. For example, the 0.91 in column (5) represents the 0.91-year increase in male HLE that would have occurred if the DFR had remained at the 1994 level because of a decrease in mortality.

**Table 2:** The effect of changes of the mortality and disability-free rate of the elderly on HLE from 1994 to 2014.

**Male**						
**Age**	**1994** **(1)**	**2004** **(2)**	**Counterfactual** **(3)**	**Δ** **(4) = (2)–(1)**	**Due to mortality** **(5) = (3)–(1)**	**Due to DFR** **(6) = (4)–(5)**
60	14.99	15.69	15.90	0.71	0.91	−0.20
65	11.42	11.96	12.14	0.55	0.72	−0.17
70	8.38	8.69	8.84	0.31	0.47	−0.16
75	6.03	6.13	6.29	0.10	0.25	−0.16
80	4.23	4.28	4.46	0.05	0.23	−0.18
85+	2.91	2.84	3.05	−0.06	0.14	−0.20
**Female**						
**Age**	**1994** **(1)**	**2004** **(2)**	**Counterfactual** **(3)**	**Δ** **(4) = (2) – (1)**	**Due to mortality** **(5) = (3) – (1)**	**Due to DFR** **(6) = (4) – (5)**
60	16.97	17.25	17.48	0.29	0.52	−0.23
65	13.13	13.32	13.52	0.19	0.39	−0.19
70	9.63	9.76	9.93	0.13	0.30	−0.16
75	6.77	6.84	6.98	0.07	0.21	−0.14
80	4.46	4.40	4.63	−0.07	0.17	−0.24
85+	2.88	2.78	3.04	−0.10	0.16	−0.26

Δ, *the change in the indicator in 1994–2004*.

The difference between the actual change and the counterfactual change in column (4) is usually smaller for the former than for the latter, indicating that HLE may be much higher if the health status of older people remains the same as in 1994. For example, if there had been no change in DFR, HLE would have risen by 0.91 years for women, compared to an actual rise of 0.71 years. Thus, the difference between 0.91 and 0.71 years indicates the effect of deteriorating health status.

The rise in mortality-induced HLE decreases with age, showing a similar age pattern to LE, HLE and its changes. Even so, almost all age groups experienced mortality improvements from 1994 to 2004, except for women over 80 years of age. DFR-induced HLE loss is usually high at age 60 and then declines. Although there are clear gender differences in longevity and health status, the benefits of declining mortality are much greater for men than for women. In addition, as shown in column (6), women suffered more health deterioration than men.

### The 2010–2015 Period

#### The Trajectory of Healthy Life Expectancy of the Elderly in China

From 2010 to 2015, both LE and HLE increased among older people in China. As shown in Table 3, the rise in LE ranged from 0.10 to 0.56 years. In addition, HLE increased by 0.12 to 0.60 years for all age groups. Even for the oldest old, the HLE increased by about 0.12–0.16 years.

**Table 3:** Disability–free life expectancy of the elderly in 2010 and 2015.

**Male**									
		**LE**			**HLE**			**HLE/LE (%)**	
Age	2010	2015	Δ	2010	2015	Δ	2010	2015	Δ
60	17.79	18.17	+0.38	17.27	17.67	+0.40	97.05	97.22	+0.17
65	14.04	14.36	+0.32	13.52	13.86	+0.34	96.27	96.53	+0.26
70	10.78	11.03	+0.25	10.26	10.54	+0.28	95.14	95.53	+0.39
75	8.17	8.37	+0.20	7.64	7.87	+0.23	93.53	94.02	+0.49
80	6.01	6.16	+0.15	5.47	5.65	+0.18	90.95	91.71	+0.76
85+	4.51	4.61	+0.10	3.96	4.07	+0.12	87.72	88.27	+0.55
**Female**									
		**LE**			**HLE**			**HLE/LE (%)**	
Age	2010	2015	Δ	2010	2015	Δ	2010	2015	Δ
60	21.45	22.02	+0.56	20.51	21.12	+0.60	95.63	95.93	+0.30
65	17.32	17.81	+0.49	16.38	16.91	+0.53	94.60	94.97	+0.37
70	13.55	13.96	+0.42	12.61	13.06	+0.45	93.12	93.56	+0.44
75	10.36	10.70	+0.34	9.42	9.78	+0.36	90.96	91.46	+0.50
80	7.64	7.90	+0.26	6.69	6.97	+0.27	87.61	88.18	+0.57
85+	5.62	5.80	+0.18	4.69	4.84	+0.16	83.39	83.55	+0.16

The increase in HLE exceeded the increase in LE, except for women over 85 years of age. It is also clear that women have an advantage over their male counterparts in terms of growth in both HLE and LE at all ages. Thus, the ratio of HLE to LE increased from 2010 to 2015, indicating morbidity compression.

#### The Effect of Mortality and Disability-Free Rate on the Changes in HLE

We applied the counterfactual analysis to the change in HLE from 2010 to 2015. Again, we calculated a hypothetical HLE using the 2010 DFR, but with two actual life tables in 2010 and 2015, and compared this to the actual change in HLE in 2010. The results are presented in Table 4.

**Table 4:** The effect of changes of the mortality and disability-free rate of the elderly on HLE from 2010 to 2015.

**Male**						
**Age**	**2010** **(1)**	**2015** **(2)**	**Counterfactual** **(3)**	**Δ** **(4) = (2)–(1)**	**Due to mortality** **(5) = (3)–(1)**	**Due to DFR** **(6) = (4)–(5)**
60	17.27	17.67	17.63	0.40	0.36	0.04
65	13.52	13.86	13.81	0.34	0.29	0.05
70	10.26	10.54	10.49	0.28	0.23	0.05
75	7.64	7.87	7.82	0.23	0.18	0.05
80	5.47	5.65	5.60	0.18	0.13	0.05
85+	3.96	4.07	4.05	0.12	0.09	0.03
**Female**						
**Age**	**2010** **(1)**	**2015** **(2)**	**Counterfactual** **(3)**	**Δ** **(4) = (2) – (1)**	**Due to mortality** **(5) = (3)–(1)**	**Due to DFR** **(6) = (4)–(5)**
60	20.51	21.12	21.02	0.60	0.51	0.10
65	16.38	16.91	16.82	0.53	0.44	0.09
70	12.61	13.06	12.98	0.45	0.37	0.08
75	9.42	9.78	9.72	0.36	0.29	0.07
80	6.69	6.97	6.91	0.27	0.22	0.05
85+	4.69	4.84	4.83	0.16	0.15	0.01

Unlike the period 1994–2004, when deteriorating health partially offset the positive impact of declining mortality, the period 2010–2015 witnessed a reversal of the DFR contributing to an increase in HLE, albeit of a modest magnitude. This reversal strongly suggests that older people have made considerable progress in improving their health since 2010. For example, the 0.40-year rise in HLE was due to 0.36 years from mortality improvements and 0.04 years from health improvements.

From 2010–2015, declines in mortality dominated the rise in HLE, although this was accompanied by an increase in the importance of health improvements. For example, declines in mortality accounted for 90% of the rise in HLE at age 60 and improvements of activities of daily life contributed to the rest 10% of the rise in HLE.

### Comparison Between 1994–2004 and 2010–2015

**Figure 1:**
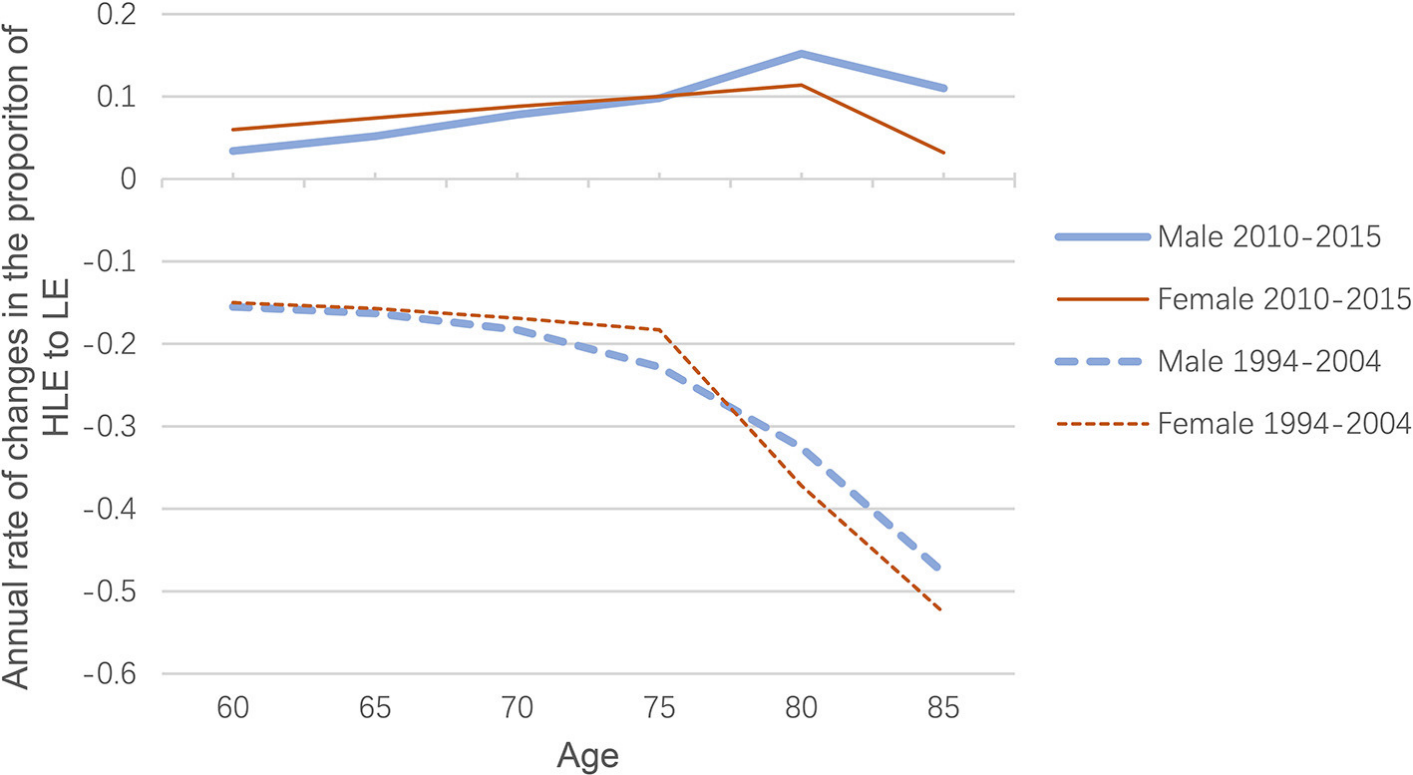
Age-specific rate of annual change in the proportion of HLE to LE for both sexes, 1994-2004 and 2010-2015.

Due to the different lengths of the two periods studied, the annual rate of exponential changes in the proportion of HLE to LE should be calculated for comparison purposes. As shown in Figure 1, blue represents males, indicating that the annual rate of change in the ratio of HLE to LE from 2010 to 2015 is much higher than the ratio from 1994 to 2004. The ratio of HLE to LE for females is the same case (red). In both periods, both sexes show similar rates of improvement in HLE relative to LE, as shown by the solid and dashed lines almost overlapping each other.

**Figure 2:**
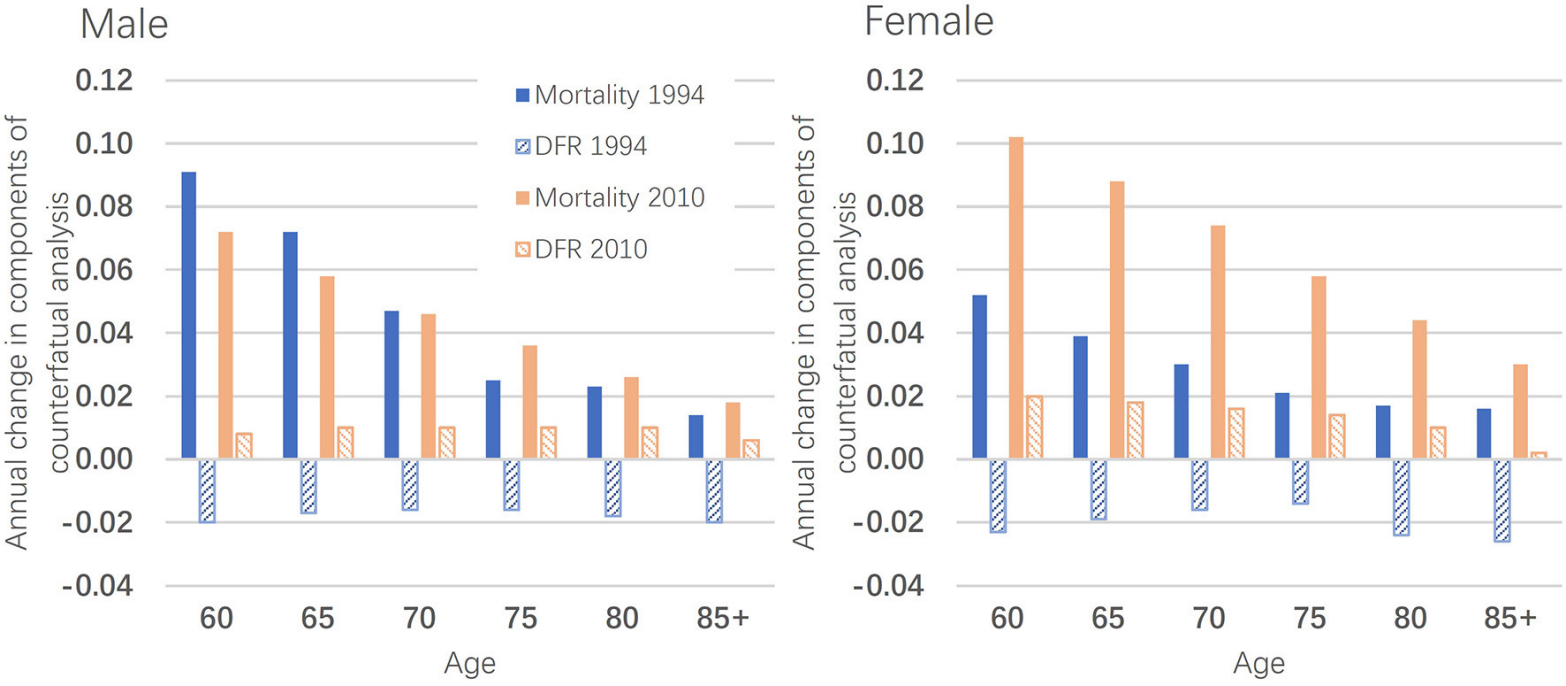
Annual change in components of counterfactual analysis.

There is clearly a transition from expansion to compression in morbidity, with the underlying factors changing over time. As shown in Figure 2, from 1994 to 2004, the increase in HLE was driven only by a decline in mortality, particularly in the early years, with the decline in DFR (disability-free rate) even offsetting the positive impact of mortality changes. However, in 2010–2015, the change in DFR has become positive and increasingly important, contributing to the rise in HLE. That is, the impact of the DFR component reversed between the 1994–2004 period and the 2010–2015 period. This reversal has brought about a shift in morbidity, although improvements in mortality have been dominating changes in HLE.

## Discussion

We examined national trends in HLE among older people in China since the mid-1990s. Based on national-level data, this study found that LE and HLE in older Chinese have steadily increased, but that the changes in HLE have created different morbidity trends. From 1994 to 2004, the proportion of HLE to LE declined, supporting morbidity expansion. However, from 2010 to 2015, the proportion increased, indicating morbidity compression. Between these two periods, a transition from expansion to compression of morbidity occurred.

A counterfactual analysis was used to explore the mechanisms underlying the morbidity transition. The increase in HLE was dominated by a decline in mortality, particularly from 1994 to 2004, with the decline in DFR even offsetting the positive impact of mortality reductions. As a result, a typical morbidity expansion occurred during this period. However, in 2010–2015, the DFR turned positive, contributing to an increase in HLE, which led to morbidity compression. The significant improvement in HLE is partly attributable to intensive tools that can reduce the demands on dependent living. With the help of these tools, some dependent older adults can regain their independence (29).

In the absence of mortality and morbidity data for the period 2004–2010, we cannot directly examine the trajectory of HLE in the period, which should mark the transition from expansion to compression of morbidity. Even so, it is reasonable to speculate that there must have been some important progress in health improvement around 2004.

China's major health reform from 2003 may be one notable development that has helped improve the health of older people. Since the launch of market-based economic reforms in 1978, the Cooperative Medical Scheme (CMS)—a community-based health care financing system that operated in the 1970s—is dismantling due to a lack of financial support from the rural collective economy (36). As a result, nearly 80 per cent of the rural population (~640 million) did not have any health insurance in 2003 (37), and health care costs have risen sharply in tandem with market-oriented health care reforms since the 1990s (38, 39). It is estimated that ~44.4% of rural older people did not have access to adequate health care in 2003 (37). Although some health care coverage is available to older people in urban areas, the vast majority of older people in urban areas still have to pay a significant amount of their own health care costs. Older people are less likely to receive adequate treatment due to limited income or lack of access to adequate health resources due to lack of health coverage (17, 20, 40). Consequently, life expectancy increases while overall health declines (16, 25, 26), leading to expanded morbidity.

In response to popular dissatisfaction with the health system, China launched a nationwide programme in rural China in 2003 known as the New Cooperative Medical Scheme (NCMS), a voluntary government-run insurance scheme that focuses on coverage for catastrophic illnesses (41). Studies have found that NCMS increases the use of resources (42). Although NCMS did not significantly impact mortality, NCMS significantly improved activities of daily living and cognitive function among older enrollees (43). In addition, participation in NCMS promotes self-rated quality and health changes in older adults (44). Importantly, the cumulative impact of health reform on older people's health implies that the positive effects of NCMS can sustain health promotion. Thus, the positive effects of health system reform can partially explain the health improvements and morbidity compression from 2010 to 2015.

There are some limitations to this study that require further research. Firstly, the 1994 and 2004 data are from large-scale surveys, which, while having nationally representative samples, are not fully comparable with the 2010 census and 2015 micro-census data. In addition, there are some differences between the survey and census questions on health status. Recognizing the issue of comparability, we focused our analysis on the relative change in morbidity indicators and limited the comparison to the relative rate of change. In general, survey participants are likely to be in better health than the population as a whole, partly because surveys do not usually include older people living in institutions and hospitals, who tend to be in poorer health than those living in communities. In addition, healthy older people are more likely to participate in surveys, producing a selective sample. Even so, the transition from expansion to compression in morbidity observed between 1994–2004 and 2010–2015 may confirm the transition in China during this period. Secondly, a long-term time series of mortality and morbidity data is desirable for studying morbidity transitions, as it can cover possible multi-stage transitions and identify their turning points. However, such data are not currently available. Furthermore, the lack of data for 2004–2010 prevents us from examining how morbidity transitioned from expansion to compression during this period. In the future, we can apply new methods to reconstruct historical data to create a complete picture of the morbidity transition in China.

## Data Availability Statement

Publicly available datasets were analyzed in this study. This data can be found here: http://www.stats.gov.cn/tjsj/pcsj/.

## Author Contributions

ZZ and QL: designed the study. ZZ, QL, JD, and CZ: revised the report, did the analysis, and interpreted the data. ZZ and QL: wrote the first draft. All authors contributed to the article and approved the submitted version.

## Funding

This study was funded by National Social Science Fund of China (21BRK024), the Ministry of Education of Humanities and Social Science Project of China (21YJA840010) and Shanghai Planning Office of Philosophy and Social Science (2020BSH014 and 2019BSH002).

## Conflict of Interest

The authors declare that the research was conducted in the absence of any commercial or financial relationships that could be construed as a potential conflict of interest. The reviewer WC declared a shared affiliation with the author JD to the handling editor at time of review.

## Publisher's Note

All claims expressed in this article are solely those of the authors and do not necessarily represent those of their affiliated organizations, or those of the publisher, the editors and the reviewers. Any product that may be evaluated in this article, or claim that may be made by its manufacturer, is not guaranteed or endorsed by the publisher.
