# Supplementary material for: Trends of Healthy Life Expectancy of the Elderly in China in 1994–2015: Revisiting From the Perspective of Morbidity Transition
Source: Front Public Health. 2022 Jan 5;9:774205. doi: 10.3389/fpubh.2021.774205 (PMC8766505; doi:10.3389/fpubh.2021.774205)
Supplement: Supplementary file 1 [file Data_sheet_1.docx]

**Tables for calculation of LE an HLE**

**Male，1994**

| Age | lx | Lx | Tx | LE | $\pi_{x}$ | Lx,DF | Tx,DF | HLE | HLE/LE |
| --- | --- | --- | --- | --- | --- | --- | --- | --- | --- |
| 60-64 | 80051 | 382249 | 1294927 | 16.18 | 0.98 | 374328 | 1199587 | 14.99 | 92.64 |
| 65-69 | 72277 | 333353 | 912678 | 12.63 | 0.96 | 320404 | 825259 | 11.42 | 90.42 |
| 70-74 | 60273 | 261026 | 579325 | 9.61 | 0.93 | 241646 | 504855 | 8.38 | 87.15 |
| 75-79 | 43617 | 173998 | 318300 | 7.30 | 0.88 | 152298 | 263209 | 6.03 | 82.69 |
| 80-84 | 26229 | 94441 | 144301 | 5.50 | 0.80 | 75101 | 110911 | 4.23 | 76.86 |
| 85+ | 12320 | 49861 | 49861 | 4.05 | 0.72 | 35811 | 35811 | 2.91 | 71.82 |

**Male，2004**

| Age | lx | Lx | Tx | LE | $\pi_{x}$ | Lx,DF | Tx,DF | HLE | HLE/LE |
| --- | --- | --- | --- | --- | --- | --- | --- | --- | --- |
| 60-64 | 85107 | 410161 | 1466267 | 17.23 | 0.97 | 397857 | 1335573 | 15.69 | 91.09 |
| 65-69 | 78375 | 367133 | 1056106 | 13.48 | 0.95 | 349912 | 937716 | 11.96 | 88.79 |
| 70-74 | 67654 | 299053 | 688973 | 10.18 | 0.92 | 274279 | 587804 | 8.69 | 85.32 |
| 75-79 | 51127 | 206512 | 389921 | 7.63 | 0.86 | 178539 | 313525 | 6.13 | 80.41 |
| 80-84 | 31545 | 116510 | 183409 | 5.81 | 0.77 | 90132 | 134985 | 4.28 | 73.60 |
| 85+ | 15778 | 66899 | 66899 | 4.24 | 0.67 | 44853 | 44853 | 2.84 | 67.05 |

**Male，2010**

| Age | lx | Lx | Tx | LE | $\pi_{x}$ | Lx,DF | Tx,DF | HLE | HLE/LE |
| --- | --- | --- | --- | --- | --- | --- | --- | --- | --- |
| 60-64 | 85005 | 409714 | 1512380 | 17.79 | 0.99 | 406209 | 1467754 | 17.27 | 97.05 |
| 65-69 | 78514 | 368607 | 1102666 | 14.04 | 0.99 | 363173 | 1061545 | 13.52 | 96.27 |
| 70-74 | 68086 | 303108 | 734059 | 10.78 | 0.97 | 295304 | 698372 | 10.26 | 95.14 |
| 75-79 | 52733 | 220533 | 430951 | 8.17 | 0.96 | 211686 | 403068 | 7.64 | 93.53 |
| 80-84 | 35004 | 129861 | 210418 | 6.01 | 0.93 | 120714 | 191381 | 5.47 | 90.95 |
| 85+ | 17864 | 80557 | 80557 | 4.51 | 0.88 | 70667 | 70667 | 3.96 | 87.72 |

**Male，2015**

| Age | lx | Lx | Tx | LE | $\pi_{x}$ | Lx,DF | Tx,DF | HLE | HLE/LE |
| --- | --- | --- | --- | --- | --- | --- | --- | --- | --- |
| 60-64 | 86116 | 416190 | 1564989 | 18.17 | 0.99 | 412540 | 1521520 | 17.67 | 97.22 |
| 65-69 | 80003 | 377056 | 1148799 | 14.36 | 0.99 | 371728 | 1108980 | 13.86 | 96.53 |
| 70-74 | 69949 | 313018 | 771743 | 11.03 | 0.98 | 305966 | 737252 | 10.54 | 95.53 |
| 75-79 | 54809 | 230859 | 458726 | 8.37 | 0.96 | 222312 | 431286 | 7.87 | 94.02 |
| 80-84 | 36985 | 138611 | 227867 | 6.16 | 0.94 | 130186 | 208974 | 5.65 | 91.71 |
| 85+ | 19353 | 89255 | 89255 | 4.61 | 0.88 | 78788 | 78788 | 4.07 | 88.27 |

**Female, 1994**

| Age | lx | Lx | Tx | LE | $\pi_{x}$ | Lx,DF | Tx,DF | HLE | HLE/LE |
| --- | --- | --- | --- | --- | --- | --- | --- | --- | --- |
| 60-64 | 85202 | 412814 | 1621870 | 19.04 | 0.97 | 402309 | 1445618 | 16.97 | 89.13 |
| 65-69 | 79468 | 377663 | 1209057 | 15.21 | 0.95 | 359987 | 1043309 | 13.13 | 86.29 |
| 70-74 | 70981 | 323204 | 831394 | 11.71 | 0.91 | 293912 | 683322 | 9.63 | 82.19 |
| 75-79 | 57520 | 246612 | 508189 | 8.83 | 0.84 | 207931 | 389410 | 6.77 | 76.63 |
| 80-84 | 40649 | 157111 | 261578 | 6.44 | 0.74 | 116861 | 181479 | 4.46 | 69.38 |
| 85+ | 22432 | 104466 | 104466 | 4.66 | 0.62 | 64618 | 64618 | 2.88 | 61.86 |

**Female, 2004**

| Age | lx | Lx | Tx | LE | $\pi_{x}$ | Lx,DF | Tx,DF | HLE | HLE/LE |
| --- | --- | --- | --- | --- | --- | --- | --- | --- | --- |
| 60-64 | 88627 | 431483 | 1745062 | 19.69 | 0.97 | 416381 | 1529252 | 17.25 | 87.63 |
| 65-69 | 83530 | 399046 | 1313579 | 15.73 | 0.94 | 376668 | 1112871 | 13.32 | 84.72 |
| 70-74 | 75419 | 346123 | 914533 | 12.13 | 0.90 | 311072 | 736203 | 9.76 | 80.50 |
| 75-79 | 62159 | 268825 | 568409 | 9.14 | 0.85 | 228432 | 425131 | 6.84 | 74.79 |
| 80-84 | 44738 | 174865 | 299584 | 6.70 | 0.72 | 126114 | 196699 | 4.40 | 65.66 |
| 85+ | 25384 | 124720 | 124720 | 4.91 | 0.57 | 70584 | 70584 | 2.78 | 56.59 |

**Female, 2010**

| Age | lx | Lx | Tx | LE | $\pi_{x}$ | Lx,DF | Tx,DF | HLE | HLE/LE |
| --- | --- | --- | --- | --- | --- | --- | --- | --- | --- |
| 60-64 | 90883 | 445112 | 1949649 | 21.45 | 0.99 | 441106 | 1864460 | 20.51 | 95.63 |
| 65-69 | 86886 | 419289 | 1504537 | 17.32 | 0.98 | 412788 | 1423355 | 16.38 | 94.60 |
| 70-74 | 80119 | 373494 | 1085248 | 13.55 | 0.97 | 363157 | 1010567 | 12.61 | 93.12 |
| 75-79 | 68701 | 306611 | 711754 | 10.36 | 0.95 | 292446 | 647410 | 9.42 | 90.96 |
| 80-84 | 53022 | 216164 | 405143 | 7.64 | 0.91 | 197373 | 354964 | 6.69 | 87.61 |
| 85+ | 33633 | 188979 | 188979 | 5.62 | 0.83 | 157591 | 157591 | 4.69 | 83.39 |

**Female, 2015**

| Age | lx | Lx | Tx | LE | $\pi_{x}$ | Lx,DF | Tx,DF | HLE | HLE/LE |
| --- | --- | --- | --- | --- | --- | --- | --- | --- | --- |
| 60-64 | 92249 | 452813 | 2030873 | 22.02 | 0.993 | 449515 | 1948267 | 21.12 | 95.93 |
| 65-69 | 88619 | 429037 | 1578060 | 17.81 | 0.988 | 423686 | 1498751 | 16.91 | 94.97 |
| 70-74 | 82298 | 385617 | 1149023 | 13.96 | 0.977 | 376854 | 1075066 | 13.06 | 93.56 |
| 75-79 | 71368 | 320885 | 763406 | 10.70 | 0.960 | 307985 | 698211 | 9.78 | 91.46 |
| 80-84 | 56010 | 230955 | 442521 | 7.90 | 0.924 | 213453 | 390227 | 6.97 | 88.18 |
| 85+ | 36496 | 211566 | 211566 | 5.80 | 0.836 | 176774 | 176774 | 4.84 | 83.55 |
